# Cardiac shock wave therapy and myocardial perfusion in severe coronary artery disease

**DOI:** 10.1007/s00392-015-0853-0

**Published:** 2015-04-17

**Authors:** M. Kaller, L. Faber, N. Bogunovic, D. Horstkotte, W. Burchert, Oliver Lindner

**Affiliations:** Institute of Radiology, Nuclear Medicine and Molecular Imaging, Heart and Diabetes Center North Rhine-Westphalia, University Hospital of the Ruhr-University Bochum, Georgstr. 11, 32545 Bad Oeynhausen, Germany; Clinic for Cardiology, Heart and Diabetes Center North Rhine-Westphalia, University Hospital of the Ruhr-University Bochum, Bad Oeynhausen, Germany

**Keywords:** Coronary artery disease, Cardiac shock wave therapy, Refractory angina, Myocardial perfusion imaging, PET

## Abstract

**Background:**

Ultrasound guided cardiac shock wave therapy (CSWT) is a noninvasive therapeutic option in the treatment of chronic-refractory angina. Clinical trials have shown that CSWT reduces angina symptoms, improves regional systolic function, LV ejection fraction, myocardial perfusion and quality of life parameters. Absolute measurements of myocardial perfusion before and after CSWT have not been performed so far.

**Methods and results:**

We studied a total of 21 CCS III patients with history of CAD and multiple interventions who suffered from disabling angina despite individually optimized medical therapy. An N-13 NH_3_ PET perfusion scan under adenosine was performed before and after CSWT treatment. CSWT was well tolerated in all patients. Absolute perfusion under adenosine of the global left-ventricular myocardium did not change under therapy or minimal coronary resistance. The treated segments, however, showed in terms of both perfusion and resistance a mild but significant improvement, by 11 and 15 %, respectively, whereas no change could be observed in the remote segments. Considering a threshold of increased perfusion of 5 %, 10 (77 %) out of 13 patients with a better target perfusion improved in their CCS class, whereas 3 (43 %) out of 7 patients without improved target perfusion improved in their CCS class too.

**Conclusion:**

Standard CSWT has the potential to improve myocardial perfusion of the therapy zone and clinical CAD symptomatology without affecting global myocardial perfusion. As a noninvasive and well tolerated therapeutic option, these data suggest the use of CSWT in patients with end-stage CAD.

## Introduction

Coronary artery disease (CAD) is one of the leading causes of morbidity and mortality in industrialized countries. Several therapeutic options for chronic CAD are available. They include medical treatment, percutaneous coronary intervention (PCI), and coronary artery bypass surgery. Despite excellent interventional results, an increasing number of patients with advanced CAD not suitable for any intervention suffer from chronic angina inspite of optimal medical therapy [1, 2]. It has been shown that persistent angina symptoms are associated with long-term anxiety, depressions, impaired physical functioning and QOL [3]. Therefore, several alternative therapeutic approaches, including epidural spinal cord stimulation (SCS) [4], enhanced external counterpulsation (EECP) [5], transmyocardial laser revascularization (TMR) [6], or induction of angiogenesis by protein, gene stem cell-based therapies [7] have been proposed. These alternatives are invasive, still at a preclinical stage, or already outdated.

Ultrasound guided cardiac shock wave therapy (CSWT) is another noninvasive therapeutic option in the treatment of chronic-refractory angina. Physically, a shock wave pulse is a single sharp pressure pulse with amplitude up to 100 MPa, followed by a tensile part of several microseconds with lower amplitude.

Animal studies have suggested that CSWT promoted angiogenesis in ischemic myocardium by mRNA expression of vascular endothelial growth factor (VEGF), endothelial cell proliferation, and endothelial nitric oxide synthase (eNOS) expression [8, 9]. Furthermore, nonenzymatic nitric oxide production by CSWT could be demonstrated [10].

Clinical trials demonstrated that CSWT may reduce angina symptoms, improve regional systolic function, LV ejection fraction, myocardial blood flow and quality of life parameters [11–14].

In this context myocardial perfusion has been evaluated with SPECT imaging at rest [15], under low-dose dobutamin stimulation [11, 13], on exercise [16] and under dipyridamole-induced hyperemia [17], all without absolute quantification. The present study evaluated with N-13 NH_3_ PET perfusion imaging under adenosine induced hyperemia the effects of CSWT treatment on absolute perfusion in the target region and in the entire left myocardium in patients with chronic-refractory angina.

## Methods

### Patient population and study protocol

We studied a total of 21 patients with history of CAD and multiple prior interventions (PCI, CABG, or both) who suffered from disabling angina despite individually optimized medical therapy (Table 1).

**Table 1:** Patient characteristics

Age	65 ± 10 years	
Gender	13 m, 8 f	
Diabetes	11 patients	
Hypertension	17 patients	

Cardiac history	No. of patients	CAD status
CAD	21	1 with 1-, 20 with 3-vessel disease
Prior infarction	11	7 with 1, 4 with 2 MI
Prior PCI	16	5 with 1, 6 with 2, 5 with ≥3 PCI
Prior CABG	19	15 with 1, 4 with 2 CABG

An N-13 NH_3_ PET scan at rest and under adenosine was performed for clinical reasons to reevaluate a repeat mechanical revascularization. If reintervention had been found inappropriate, the viable myocardial wall with the leading perfusion abnormality was identified for CSWT. The baseline PET scan was performed 21 ± 36 days before onset of therapy. A repeat PET scan was performed 43 ± 27 days after the last CSWT session for clinical reasons, to verify the therapeutic effect on regional perfusion and to decide upon continuation of therapy.

Canadian Cardiovascular Society (CCS) scale was assessed in all patients before and after therapy. Additionally, echocardiography and, if feasible, ergometry were performed.

Patients gave their written informed consent to CSWT and the clinical PET studies. The data analysis was approved by the local ethics committee of the Ruhr-University Bochum (Reg. No. 60/2013).

### Image acquisition and stress testing

Patients were investigated with an ECAT-951 R scanner (CTI/Siemens Medical systems) and, after a change of the device, with a Biograph mCT (Siemens, Erlangen, Germany). Directly before the PET acquisition, a transmission scan was performed with an ECAT-951 R scanner and a low-dose CT with the Biograph mCT for attenuation correction.

Adenosine was infused intravenously at a constant rate of 0.14 mg/kg/min over 6 min. During the stress phase, heart rate and blood pressure were recorded every 2 min, starting with the onset of the adenosine infusion until completion of after 6 min. Mean arterial blood pressure was calculated from the average values of all four time points and minimal coronary resistance as mean arterial blood pressure/global perfusion.

Two minutes after the onset of the adenosine infusion, on average 600 MBq N-13 NH_3_ were injected as an intravenous bolus. Image acquisition over 15 min was started simultaneously with the bolus injection. A consecutive set of 20 frames was reconstructed for quantification of perfusion.

### Quantitative perfusion analysis

Quantification of the N-13 NH_3_ scans was based on an irreversible two-compartment model [18]. Corrections for fractional blood volume, partial volume effects and spillover activity from left-ventricular blood pool to tissue were calculated as described elsewhere [19]. A validation of the model in humans had been performed by the argon inert gas technique beforehand [20]. The quantification procedure delivered 20-segment parametric polar maps of MBF. Segments with a fractional blood volume >0.5 were excluded from further analysis. Such large values are only explainable by spillover from the ventricle and mostly referred to the basal segments of the septum. Furthermore, segments with a resting MBF < 50 mL/min/100 g were regarded as scarred and also excluded from the analysis. Global perfusion was calculated as the average of all myocardial segments [21].

The CSWT target zone was assigned to the anteroseptal wall in 8 cases, to the inferolateral wall in 6, to the inferior wall in 6, and to the apex in 1 case. The reference wall was defined as the remote wall opposite to the CSWT target zone. In the case of the apical CSWT target, the anterior wall served as the reference. For quantitative analysis, perfusion values of the segments of the parametric polarmaps representing either the target or the remote wall were averaged.

### Shock wave therapy device, procedure and protocol

A detailed description with illustration of the device and the CSWT protocol has been given elsewhere [13]. In brief, shockwaves were applied with a commercially available shock wave generator (Cardiospec, Medispec, Gaitersburg, USA) under echocardiographic guidance. The energy flux density was adjustable between 0.03 and 0.2 mJ/mm^2^, with a focus size of 8–9 mm and a length of 25 mm to ensure transmural coverage. The focus could be set up to a depth between 4 and 15 cm. During the procedure, patients lay in supine position. ECG, blood pressure and vital signs were monitored continuously.

The target region previously defined with the PET scan was adjusted with the ultrasound probe. Shock wave release occurred with an R-wave ECG trigger.

The CSWT scheme was performed over 3 months (93 ± 34 days) in blocks of 3 sessions applied every other day during the treatment weeks, with 6 weeks intervals (44 ± 25 and 42 ± 15 days, respectively). The target region previously defined with PET was divided into a basal, midcavity, and apical zone. During each treatment, these zones were targeted with the ultrasound probe, progressing from the basal zone in week 1 (sessions 1–3) to the midcavity zone in week 2 (sessions 4–6), and the apical zone in week 3 (sessions 7–9). Patients received 300 shocks (when starting) to 500 shocks (after verification of tolerance) per treatment session. Thus, during the 9 shockwave sessions, a total of 2700–4500 individual discharges were delivered. Troponin I was controlled several hours after each application.

### Echocardiography

Resting two-dimensional echocardiography was performed at baseline and follow-up with a 1.7/3.3 MHz multifrequency probe in harmonic imaging mode using commercially available echo equipment (Vivid E9, General Electric, Horten, Norway). The classical two apical views (2- and 4-chamber views) were applied to measure biplane LVEF and both the endsystolic and enddiastolic volumes.

### Exercise stress testing

The bicycle test was performed in the upright position (Ergometer 900 ERG, GE Medical Systems). Depending on the patient’s condition, ergometry was started at 25 W or 50 W, and was increased by 25 W each minute. The test was stopped when typical chest pain, significant ST depressions, or exhaustion of the leg muscles occurred.

### Statistical analysis

For the sample size estimation, a power of 90 % and a significance criterion of 0.05 were chosen. The minimum expected difference between the two means and the standard deviation were estimated to 10 mL/min/100 g each. Accordingly, about 20 patients had to be enrolled [22].

Data are given as mean value ± standard deviation. In the first step, the paired parameters were tested for normal distribution with the Kolmogorov–Smirnov test. As all parameters were normally distributed, post hoc comparisons were performed with a paired *t* test.

The contingency between change in CCS class (unchanged or better) and change in perfusion (increase <5 % and increase ≥5) was examined using Fisher’s exact test.

Differences were considered statistically significant at values <0.05 (two-sided). For the analyses, the statistical software package IBM SPSS (version 20) was used.

## Results

### Clinical characteristics, medication, and clinical parameters

The baseline clinical characteristics of the patients and their cardiac medication are shown in Tables 1 and 2. Neither dosages of long-acting nitrates nor the other cardiac medicaments were changed during the observation period.

**Table 2:** List of cardiac medication

	No. of patients
Aspirin	14
Clopidogrel	7
β-blockers	21
ACE inhibitors/AT1-receptor antagonists	19
Diuretics	13
Calcium antagonists	4
Nitrates	21
Ranolazin	4
Statins	19

CSWT was well tolerated in all patients. No patient showed an increase in troponin I beyond the reference level after the applications, and no therapy course had to be stopped prematurely.

All patients were in CCS class III at baseline. At follow-up, a downstaging was observed in 13 patients, such that 13 were in CCS class II and 8 remained in CCS class III (Table 3).

**Table 3:** Target region, PET perfusion, and change in CCS class of the individual patients

Pat No.	CSWT Target	Perfusion of target segments		Perfusion of remote segments		Perfusion of the global myocardium		Delta CCS^a^
		Baseline	Follow-up	Baseline	Follow-up	Baseline	Follow-up	
1	Inferior	91	123	131	150	130	156	−1
2	Anteroseptal	143	152	97	104	139	149	0
3	Lateral	66	74	116	138	88	106	−1
4	Anteroseptal	87	71	141	107	105	75	0
5	Inferior	184	162	244	171	241	192	−1
6	Inferior	114	156	174	209	144	171	0
7	Anteroseptal	162	173	167	174	162	180	0
8	Lateral	67	146	123	154	101	152	−1
9	Lateral	192	220	152	192	170	192	−1
10	Lateral	77	81	106	96	99	94	−1
11	Apikal	230	227	326	311	284	302	0
12	Anteroseptal	116	130	90	96	96	95	−1
13	Lateral	76	93	134	150	94	120	−1
14	Anteroseptal	161	165	218	231	190	208	0
15	Anteroseptal	164	167	204	196	178	183	0
16	Inferior	76	101	79	91	94	103	−1
17	Lateral	119	135	140	117	157	161	−1
18	Anteroseptal	72	95	60	70	68	71	−1
19	Inferior	190	182	215	231	228	213	−1
20	Anteroseptal	77	92	115	99	82	81	0
21	Inferior	58	42	79	88	75	86	−1

PET perfusion values are given in mL/min/100 g

^a^−1: a change from CCS classes III to II was observed, 0: no change in CCS class

Exercise testing could be completed in 16 patients at baseline and follow-up. In 5 patients, ergometry was not feasible due to non-cardiac limitations. Mean achievable workload at baseline was 93 ± 44 W, and at follow-up 101 ± 41 W (n.s.).

### PET perfusion measurements and correlation to CCS class

**Fig. 1:**
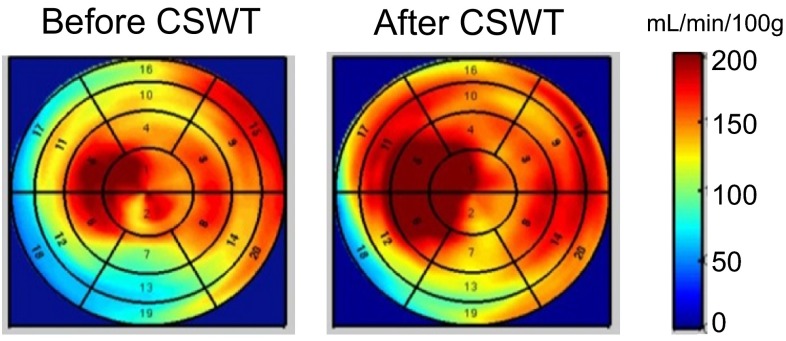
PET polartomograms of absolute perfusion under adenosine of patient 1 (Table 3) before and after CSWT. In this case, the inferior wall was treated. Perfusion of the target zone under adenosine was 91 before CSWT and 123 mL/min/100 g after therapy

The perfusion data are listed in Tables 3 and 4. Absolute myocardial perfusion of the global left-ventricular myocardium under adenosine induced hyperemia did not change under therapy, nor did minimal coronary resistance (perfusion related to perfusion). The treated segments, however, in terms of both perfusion and resistance, showed a mild but significant improvement, 11 and 15 %, respectively, whereas, no change could be observed in the remote segments. Figure 1 illustrates the PET scans of a typical case. This patient experienced a downstaging to CCS class II.

**Table 4:** PET perfusion measurements of the entire cohort

	Perfusion under adenosine (mL/min/100 g)			Minimal coronary resistance [mmHg/(mL/min/100 g)]		
	Baseline	Follow-up	*P*	Baseline	Follow-up	*P*
CSWT target segments	120 ± 52	133 ± 49	0.014	0.84 ± 0.36	0.72 ± 0.30	0.028
Remote segments	148 ± 64	151 ± 61	0.6	0.67 ± 0.31	0.66 ± 0.26	0.76
Global myocardium	139 ± 59	147 ± 59	0.1	0.80 ± 0.38	0.71 ± 0.33	0.24

Considering a threshold of improvement in perfusion of 5 %, based on an estimated error of measurement of 5 %, 10 (77 %) patients out of 13 with a better perfusion of the target segments improved in their CCS class, whereas, 3 (43 %) patients out of 7 without better perfusion of the target segments improved in their CCS class, too (Table 5). This 5 % value was based on the general variability of repeated PET N-13 ammonia measurements under adenosine [23].

**Table 5:** CCS class and perfusion data

	CCS unchanged	CCS improved
Perfusion shift <5 %	5	3
Perfusion shift ≥5 %	3	10

*P* = 0.16

### Echocardiographic measurements

The results are given in Table 6. No significant changes in the global left-ventricular parameters enddiastolic diameter (LVEDD), enddiastolic (LVEDV), endsystolic (LVESV) and ejection fraction (LVEF) were observed between baseline and follow-up.

**Table 6:** Echocardiographic measurements

	Baseline	Follow-up	*P*
LVEDD (mm)	55 ± 7	53 ± 6	0.36
LVEDV (ml)	91 ± 34	96 ± 25	0.46
LVESV (ml)	47 ± 33	49 ± 24	0.29
LVEF (%)	54 ± 17	51 ± 14	0.74

*LVEDD* left-ventricular enddiastolic diameter, *LVEDV* left-ventricular enddiastolic volume, *LVESV* left-ventricular endsystolic volume, *LVEF* left-ventricular ejection fraction

## Discussion

CSWT considers an alternative therapeutic approach in the treatment of patients with end-stage CAD without any further interventional option, but with angina symptoms despite optimized individual medication. Although the exact mechanism of CSWT in myocardial tissue is not completely understood, the main effect seems to be a local stimulation of neoangiogenesis. This may be induced by two pathways: (1) increase of local shear stress by the collapse of microbubbles, and (2) stimulation of local angiogenesis by angiogenesis-related growth factors and recruitment of endothelial progenitor cells [8, 9, 24].

Several studies demonstrated that CSWT is able to improve clinical symptoms, and quality of life parameters [11, 12, 14]. Evaluations have been performed with several nuclear imaging techniques. They showed, on a qualitative basis of imaging analysis, an improvement in resting perfusion in 14 patients and exercise perfusion in 10 patients [15, 16]. A more dedicated analysis in 9 patients demonstrated an improvement in the treated area, while the untreated area tended to worsen [17]. One study failed to show significant differences in myocardial perfusion in 9 patients at rest and under low-dose dobutamine stimulation [11].

In the present study, we could demonstrate a significant increase in absolute myocardial perfusion in the treated segments, whereas no change in perfusion occurred in the remote segments. Coronary resistance showed a concordant behavior indicating that the perfusion changes were not exclusively related to improvements in hemodynamics and has to be assigned to further factors directly affecting myocardial perfusion. CSWT-induced neoangiogenesis may be one potential component, but the extent to which it contributes required additional morphological studies.

Normal absolute myocardial perfusion under hyperemia is usually assumed >200 mL/min/100 g [25]. Against this background, mean global myocardial perfusion under adenosine was severely diminished in our patient cohort, but within the range of hyperemic perfusion in ischemic heart disease [26]. The relative high standard deviation of the individual data indicates that they extend over a long range and that the CSWT patient group itself represents a heterogeneous subset.

In terms of global myocardial perfusion and minimal coronary resistance, no significant changes were found during the 4 months between the observation periods. These results are in accordance with the echocardiographic measurements indicating that no significant change of the disease occurred during this period. Nevertheless, a treatment of the leading ischemic area seems to be beneficial with respect to the CCS classification, although no significant changes in exercise tolerance were observed. To what extent this improvement in CCS classification is a net effect of reduced ischemic burden, and thus a relief of angina symptoms, or is driven by changes in individual perception needs further clarification. The present data indicate that suggestive factors are likely to play a role as 38 % of those patients with no better perfusion of the target segments experienced a CCS class downstaging.

Another issue of interest is the duration of the improvements, with the concept for future therapeutic courses having to evaluate the frequency and duration of CSWT and, finally, patient outcome.

The results of the present study are in line with former imaging studies and demonstrate absolute effects of CSWT on myocardial perfusion of the target zone [17]. The clinical results, however, are more cautious than those of other studies, but concordant to the global perfusion analysis which indicates that the underlying end-stage CAD remains as expected and that a local noninvasive procedure may not significantly improve an advanced stage of disease [11, 13].

To summarize the results of the present study: CSWT has the potential to improve myocardial perfusion of the target therapy zone and CAD symptomatology without affecting global myocardial perfusion and morphologic ventricular parameters. As a noninvasive and well tolerated therapeutic option, our data suggest the use of CSWT in patients with end-stage CAD.

### Study limitations

From a statistical point of view, the patient number is sufficient to identify differences in perfusion with PET imaging and mildly larger than in other studies with nuclear medicine imaging. Nevertheless, larger patient cohorts with longer follow-ups are needed to further evaluate the efficacy of CSWT. A placebo-controlled group is missing. However, the intraindividual comparison of treated vs nontreated zones tried to circumnavigate this issue.
